# Ex vivo expanded natural killer cells from breast cancer patients and healthy donors are highly cytotoxic against breast cancer cell lines and patient-derived tumours

**DOI:** 10.1186/s13058-017-0867-9

**Published:** 2017-07-01

**Authors:** Mira M. Shenouda, Amy Gillgrass, Tina Nham, Richard Hogg, Amanda J. Lee, Marianne V. Chew, Mahsa Shafaei, Craig Aarts, Dean A. Lee, John Hassell, Anita Bane, Sukhbinder Dhesy-Thind, Ali A. Ashkar

**Affiliations:** 1 Department of Pathology and Molecular Medicine, McMaster Immunology Research Centre, McMaster University, 1280 Main Street West, MDCL 4015 Hamilton, ON Canada; 2 Cellular Therapy and Cancer Immunology Program, Department of Hematology/Oncology and BMT, Nationwide Children’s Hospital, The Ohio State University Comprehensive Cancer Center, Ohio, USA; 3 Department of Oncology, McMaster University, Hamilton, ON Canada

**Keywords:** Immunotherapy, PDX, NK cell expansion, NK cell therapy

## Abstract

**Background:**

Natural killer (NK) cells play a critical role in cancer immunosurveillance. Recent developments in NK cell ex-vivo expansion makes it possible to generate millions of activated NK cells from a small volume of peripheral blood. We tested the functionality of ex vivo expanded NK cells in vitro against breast cancer cell lines and in vivo using a xenograft mouse model. The study aim was to assess functionality and phenotype of expanded NK cells from breast cancer patients against breast cancer cell lines and autologous primary tumours.

**Methods:**

We used a well-established NK cell co-culture system to expand NK cells ex vivo from healthy donors and breast cancer patients and examined their surface marker expression. Moreover, we tested the ability of expanded NK cells to lyse the triple negative breast cancer and HER2-positive breast cancer cell lines MDA-MB-231 and MDA-MB-453, respectively. We also tested their ability to prevent tumour growth in vivo using a xenograft mouse model. Finally, we tested the cytotoxicity of expanded NK cells against autologous and allogeneic primary breast cancer tumours in vitro.

**Results:**

After 3 weeks of culture we observed over 1000-fold expansion of NK cells isolated from either breast cancer patients or healthy donors. We also showed that the phenotype of expanded NK cells is comparable between those from healthy donors and cancer patients. Moreover, our results confirm the ability of ex vivo expanded NK cells to lyse tumour cell lines in vitro. While the cell lines examined had differential sensitivity to NK cell killing we found this was correlated with level of major histocompatibility complex (MHC) class I expression. In our in vivo model, NK cells prevented tumour establishment and growth in immunocompromised mice. Finally, we showed that NK cells expanded from the peripheral blood of breast cancer patients show high cytotoxicity against allogeneic and autologous patient-derived tumour cells in vitro.

**Conclusion:**

NK cells from breast cancer patients can be expanded similarly to those from healthy donors, have a high cytotoxic ability against breast cancer cell lines and patient-derived tumour cells, and can be compatible with current cancer treatments to restore NK cell function in cancer patients.

## Background

Despite efforts to improve early diagnosis and treatment for breast cancer, it remains the most common type of cancer and the second leading cause of cancer-related death in women [1–3]. Several targeted therapies have been developed which have proven to be effective, but treatments for cancers such as triple negative breast cancer (TNBC) are limited. As a result, the development of effective treatments against these aggressive breast cancer subtypes are required [4].

Recently, a variety of therapies targeting breast cancer have been developed, particularly in the field of cancer immunotherapy which harnesses the power of the immune system to treat cancer. The use of natural killer (NK) cells, a type of innate lymphocyte, for adoptive cell therapy has garnered extensive interest. NK cells have the ability to recognize and lyse transformed and tumour cells without any prior sensitization and play a major role in cancer immunosurveillance [5, 6]. However, the ability of NK cells to recognize and kill tumour cells is significantly impaired in cancer patients [7–9]. The tumour microenvironment is capable of dampening NK cell activation and cytotoxicity. Tumour cells are able to recruit immunosuppressive cells such as tumour-associated macrophages, myeloid-derived suppressor cells (MDSCs) [10, 11] and regulatory T cells [12]. In a hypoxic tumour microenvironment the tumour cells secrete soluble factors that supress NK cell activity allowing the tumour to escape NK cell-mediated killing [13–15].

NK cells from cancer patients exhibit an inhibitory phenotype characterized by an increased expression of inhibitory markers, such as killer-cell immunoglobulin-like receptors (KIRs) and NKG2A, and decreased expression of activating markers, mainly NKG2D and natural cytotoxicity receptors (NCRs) [7, 9]. This inhibitory phenotype is also associated with dampened NK cell cytotoxicity when compared to NK cells isolated from healthy donors. In addition, cancer patients have a decreased number of NK cells in peripheral blood compared to healthy donors [8]. Several studies have shown that the survival rate of breast cancer patients correlated with increased NK cell expression of activating markers and that NK cell dysfunction correlated with tumour progression and invasiveness [8, 16]. Given the importance of NK cells in cancer immunosurveillance, a therapy that would allow for the delivery of a vast number of activated NK cells to cancer patients and subsequently the ability to overcome the immunosuppressive tumour environment would have great potential [15].

The use of NK cells in adoptive cell therapy has only been possible in recent years due to advances in NK cell expansion protocols [17, 18]. It is now possible to expand NK cells into billions of activated NK cells from only a few millilitres of peripheral blood. This cleared a major hurdle in the field of NK cell immunotherapy, allowing their use as an autologous cellular therapy product. Several protocols have been developed to obtain a sustainable expansion of NK cells. Initially, these protocols included the use of cytokines, such as interleukin (IL)-2 or IL-15 [19, 20], or anti-CD3 (OKT3) antibody [21]. More recently the use of irradiated feeder cells has emerged to allow for a better sustainable expansion of NK cells that was not achievable with the use of cytokines only. For this purpose, protocols either use autologous or allogenic peripheral blood mononuclear cells (PBMCs) or genetically modified tumour cells that are sensitive to NK cell killing [22–25]. These modified feeder cells express membrane-bound cytokines, antigen-presenting cell markers, and NK cell receptor ligands [17, 26, 27]. Each NK cell expansion protocol has advantages and disadvantages and yields a different fold expansion and purity of NK cells [28]. Regardless of the expansion protocol, studies have shown that expanded NK cells display an activated phenotype and increased cytotoxicity compared to NK cells freshly isolated from peripheral blood [17, 22, 29]. In our study, we adopted an extremely effective expansion protocol developed by Dr. Dean A. Lee’s group utilizing the use of artificial antigen-presenting cells (aAPCs) genetically modified to express membrane bound IL-21 [17, 30].

Clinical studies have shown great promise in the use of autologous NK cells against cancer [24, 31–33]. Infusion of large doses of NK cells into cancer patients has been shown to be safe as patients do not experience any associated severe toxic side effects [32]. In cancer patients, these expanded NK cells have been shown to survive in vivo for weeks and even months [24]. Moreover, Sakamoto et al. observed almost a threefold increase in NK cell cytotoxicity 14 days after NK cell infusion into patients who showed a stable disease state [32].

Ex-vivo expanded NK cells have enhanced cytotoxicity against K562 cells and other tumour cell lines compared to NK cells freshly isolated from healthy donors and cancer patients [22, 34, 35]. Furthermore, autologous activated NK cells do not have a toxic effect on healthy cells, thus avoiding the toxicity that is often associated with cytokine therapies [36]. Due to their safety, potent cytotoxicity against tumour cells, and the technology to extensively expand these cells, NK cells are an ideal candidate for adoptive cell cancer immunotherapy. The augmentation of NK cell activity by ex vivo expansion prior to adoptive transfer could provide a better therapeutic treatment for cancer and, as such, has been studied in clinical trials over recent years [6]. The goal of this study is to apply ex vivo expanded NK cells in an adoptive cell therapy that could be used to treat solid tumours, especially TNBC, for which treatment options are limited [37]. Here, we investigate the functionality of ex-vivo expanded NK cells against both allogeneic and autologous breast cancer cells. We aim to increase both the number and cytotoxic activity of cancer patient-derived NK cells in order to generate cellular products that can overcome the immunosuppressive tumour microenvironment. Therefore, we explored the ability to expand NK cells from breast cancer patients and compare their functionality and phenotype to those expanded from healthy donors.

## Methods

### Ethics statement

Clinical samples and peripheral blood were obtained from donors who gave written and informed consent. Sample collection was approved by the Hamilton Integrated Research Ethics Board (HIREB).

### PMBC isolation

Blood samples were collected from healthy female donors or female breast cancer patients in an ACD Solution A Vacutainer (BD; Cat. No. 364606). PBMCs were collected using a density gradient medium, Ficoll-Paque Plus (GE Healthcare Life Sciences), or Lymphoprep (StemCell). The buffy coat, containing lymphocytes, was collected and cells were counted. PBMCs were either used for immediate NK cell expansion or cryopreserved [30].

### NK cell expansion

Freshly isolated or frozen PBMCs were co-cultured with irradiated K562mbIL-21 clone9/IL-21aAPC (kindly provided by Dr. Dean A. Lee, University of Texas) feeder cells at a ratio of 1:2, respectively. Cells were cultured in complete RPMI media (10% fetal bovine serum (FBS), 1% HEPES, 1% l-glutamine, 1% penicillin-streptomycin) and 100 U/mL recombinant human IL-2 (Peprotech). Medium was changed every 2 to 3 days and co-cultures were replenished with irradiated K562mbIL-21 aAPCs on a weekly basis [17, 30].

### Flow cytometry

NK cells were stained with anti-CD3-APC-H7, anti-CD56-PE or BV-421, anti-CD16-Alexa Fluor 700, anti-CD57-APC, anti-CD25-APC or BV785, anti-CD11b-Texas Red, anti-CD27-PerCpCy5.5, anti-NKG2D-PerCpCy5.5, anti-NKp30-APC, anti-NKp-44-PE, anti-NKp46-BV786, anti-CD69-PE-Texas Red, anti-NKG2A-PE-Cy7, anti-CD158a-APC, anti-CD158b-PE, anti-HLA-A,B,C-PE, and/or anti-CD158e1-Alexa Fluor 700. Then cells were washed with FACS Buffer (0.2% bovine serum albumin (BSA)), fixed with 1% paraformaldehyde (PFA), and analysed on the BD Biosciences FACS Canto, FACS-LSR Fortessa, or FACS-LSR II. Data analysis was performed using FlowJo software (Treestar). Cells were gated on CD56^+^CD3^–^ events (NK cells) and then analysed for the expression of other extracellular surface markers.

### Staining of whole blood from human or mouse

Each blood sample was collected in an ACD Solution A Vacutainer; 100 μL of whole blood was incubated with anti- mouse CD16/CD32 antibody (eBioscience) for 20 min at room temperature (for mouse samples only). Next, an antibody cocktail was added to the blood sample for 30 min on ice. After 30 mins, 2 mL of 1× 1-step Fix/Lyse Solution (eBioscience) was added to the samples for 15 min. After the incubation, the samples were centrifuged, washed, and resuspended in FACS buffer to be analysed using flow cytometry.

### Cytotoxicity assay using breast cancer cell lines

TNBC cell line MDA-MB-231/luc or HER2-positive cell line MDA-MB-453 cells were labelled with carboxyfluorescein succinimidyl ester (CFSE; Sigma Aldrich). CFSE-labelled tumour cells were plated at 2 × 10^5^ or 4 × 10^5^ cells per well, and expanded NK cells were added and mixed in the wells. After a 4–5 h incubation at 37 °C, cells were spun down, washed, and stained with a Fixable Viability Dye, eFluor® 780 (eBioscience). Cells were subjected to extracellular staining if applicable. Samples were run on BD Biosciences FACS Canto or FACS-LSR Fortessa. Cells were gated on CFSE-labelled events and cell death was calculated based on the APC-Cy7^+^ (FVD eFluor® 780^+^) gate (dead cells). Percent-specific lysis was calculated as follows: % specific lysis = (100 × (% lysis – % basal lysis))/(100 – % basal lysis).

### Mice

NOD-Rag1^–/–^-γ^–/–^ (NRG) mice were purchased from The Jackson Laboratory and were bred at McMaster’s Central Animal Facility in accordance with standard protocols approved by McMaster’s Animal Research Ethics Board (AREB).

### In vivo NK cell injection

Ten million expanded NK cells were intravenously (IV) injected into NRG mice via the tail vein. Mice were then administered 20,000 units of IL-2 IV immediately after NK cell injection, and intraperitoneally on a daily basis after the first injection.

### Mouse tissue processing and cell isolation

#### Liver

The liver was harvested from mice, minced, and passed through a 40-μm cell strainer. Cells were spun down, resuspended in 20 mL phosphate-buffered saline (PBS) and lymphocytes were isolated using a density gradient medium. The buffy coat was collected and cells were washed, counted, and plated for staining.

#### Spleen

The spleen was harvested and minced, and then ACK lysed for 2 min. Cells were then washed, counted, and plated to be stained. We then adopted a new protocol that avoids the use of ACK lysis solution. First, we minced the spleen, counted cells, and then stained the cells with antibodies for 30 min. After staining, the cells were incubated with 2 mL of 1× 1-step Fix/Lyse Solution (eBioscience) for 15 min to eliminate red blood cells.

#### Blood

Blood was collected from mice through facial bleeding into microfuge tubes containing 150–200 μL anti-coagulant. Blood was then transferred to a 15-mL falcon tube and 2 mL of ACK lysis buffer was added for 5 min. Solutions were then diluted with PBS, spun down, and ACK lysed again in 1 mL for 1 min. Then cells were plated to be stained. Alternatively, we used the protocol for whole blood staining, described above.

#### Lungs

After euthanizing the mice, the lungs were perfused by injecting 10 mL PBS into the right chamber of the heart. Lungs were then collected and minced using blades/surgical scissors. The tissue was digested in 10 mL of 3 mg/mL Collagenase A (Roche) for 45 min. Lungs were filtered through a 40-μm cell strainer, then ACK lysed if needed. Finally, cells were washed, counted, and plated for staining.

### Patient-derived xenograft preparation

Patient samples were acquired from Henderson and St. Joseph’s Hospitals in Hamilton, Ontario. Patient samples were placed in 10% DMSO and 90% FBS and stored in liquid nitrogen. The #4 inguinal mammary fat pads of 3-week-old NRG mice, bred in-house, were humanized. This procedure involved clearing the pads and injecting them with 5 × 10^5^ human reduction mammary fibroblasts (RMF) using a Hamilton syringe (the RMF cell line was graciously provided by Dr. Charlotte Kuperwasser)—50% irradiated and 50% non-irradiated. Irradiated fibroblasts were prepared by irradiating wild-type RMF cells with 4 Gy. Two weeks after humanization, a single cell suspension of 1 × 10^5^ human breast tumour cells were injected into both the left and right humanized #4 fat pads using a Hamilton syringe. Human RMF and human breast tumour samples were injected into the mice in a solution of 50% Matrigel, 47.5% PBS, and 2.5% FBS [38].

### Cytotoxicity assay against patient-derived tumours

Breast biopsies were performed on breast cancer patients A and B, who were diagnosed with TNBC and oestrogen receptor-positive, progesterone receptor-positive, and HER2-negative (ER^+^/PR^+^/HER2^–^) breast cancer, respectively. Samples were passaged in mice as described previously. We were able to stably culture cells from breast cancer patient B and stably passage a sample in mice from patient A. For subsequent passaging in mice, the mice were euthanized at end-point based on body weight and tumour size, and tumours were collected for processing into 1-mm^2^ pieces and then injected subcutaneously into NRG mice. For in vitro cytotoxicity assays, tumour pieces were placed in 20 mL of 3 mg/mL Collagenase A (Roche) and left in a 37 °C incubator shaker for 25 min. Cells were filtered through a 40-μm cell strainer and Ficoll gradient centrifugation was performed to select for the live cell fraction. Cells were resuspended and plated in complete Dulbecco’s modified Eagle’s medium (DMEM). Peripheral blood mononuclear cells from either the same patient (autologous) or a different breast cancer patient (allogeneic) were obtained and co-cultured for NK cell expansion as previously described. After 3 weeks of co-culture, expanded NK cells were labelled with CFSE and incubated with either autologous or allogeneic tumour cells for 4–5 h. After the incubation period, cells were stained with fixable viability dye and analysed using flow cytometry. Alternatively, if expanded NK cells were not CFSE-labelled, cytotoxicity wells were stained with human anti-CD56 antibody to gate out expanded NK cells. Cell death was calculated as previously described.

### Statistical analysis

Statistical analyses were performed using GraphPad Prism 5.0 (GraphPad Software). Data are presented as mean ± SEM. A *t* test was used to compare differences between two groups. A *p* value <0.05 was considered statistically significant. A two-way analysis of variance (ANOVA) was used to compare two different variables with Bonferroni as a post-hoc test.

## Results

### Ex vivo expansion of NK cells from healthy donors leads to an increase in the expression of activation receptors and a decrease in maturation markers

**Fig. 1:**
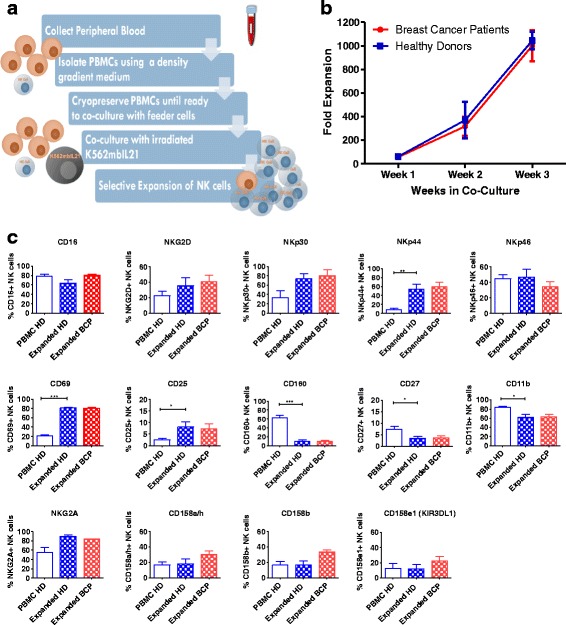
Expansion and phenotype of expanded natural killer (*NK*) cells from breast cancer patients and healthy donors. **a** A schematic showing the expansion protocol of NK cells. **b** Frozen peripheral blood mononuclear cells (*PBMCs*) from breast cancer patients (*red*) or healthy donors (*blue*) were cultured with irradiated feeder cells K562mbIL21 and 100 U/μL of IL-2. Co-culture was supplemented with IL-2 and new media every 2–3 days, and with irradiated feeder cells on a weekly basis. Cells were counted every week using trypan blue. Fold expansion was calculated assuming that NK cells comprise 15% of PBMCs (*n* = 3, mean ± SEM). Unpaired Student’s *t* test was used to compare the difference between groups. **c** Freshly isolated PBMCs from healthy donors (*HD*), expanded HD NK cells, or expanded breast cancer patient (*BCP*) NK cells were stained for the expression of CD45, CD56, CD3, CD16, NKG2D, NKp44, NKp46, NKp30, CD69, CD160, CD11b, CD27, CD25, NKG2A, CD158a, CD158b, and CD158e1. Cells were gated on a CD45^+^CD56^+^CD3^–^ population and then analysed using FlowJo Software for the expression of other markers. Pecent-positive expression of the markers was then tabulated and graphed (*n* = 3–8, mean ± SEM). Welch’s *t* test was used for statistical comparison between HD NK from freshly isolated PBMC and HD expanded NK cell groups and separately between expanded HD and BCP NK cells. *P < 0.05, **P < 0.005, ***P < 0.0005, only significant differences are indicated on graphs

Since the ex vivo expansion of NK cells has been shown to alter their phenotype, we sought to characterise the phenotype of NK cells from peripheral blood compared to those co-cultured for 3 weeks with irradiated K562mbIL-21 feeder cells (expanded NK cells) (Fig. 1a). We examined the surface expression of various activating, maturity, and inhibitory markers. Our results show that there is a significant increase in the expression of the activating receptors CD69 and NKp44 on expanded NK cells compared to those from freshly isolated PBMCs (Fig. 1c). Moreover, we saw an increase in expression of CD25 (IL2Rα). However, expanded NK cells show a decreased expression of CD11b and CD27 maturation markers that would suggest they have a less mature phenotype. Interestingly, there was a significant decrease in the expression of CD160 (Fig. 1c), an activating receptor that has been shown to be associated with interferon (IFN)-γ production, although its exact function remains largely unknown [39]. Thus, we showed that the expansion of NK cells changes their phenotype compared to NK cells freshly isolated from peripheral blood.

### NK cells expanded from breast cancer patients show similar expansion and phenotype to those expanded from healthy donors

Since NK cells from cancer patients are often deficient in their activation, we were interested in comparing the growth and phenotype of NK cells derived from healthy donors and breast cancer patients. We obtained PBMCs from breast cancer patients or healthy donors and had them cryopreserved. We performed cryopreservation prior to expansion to mimic what would likely occur if this approach was applied in the clinic. When co-culturing NK cells, we found that NK cells from both breast cancer patients and healthy donors expanded similarly to about a 1000-fold expansion by 21 days (Fig. 1b). In addition, the growth of NK cells expanded from freshly isolated or cryopreserved healthy donor PBMCs was similar to that of cells which had been cryopreserved (data not shown). Moreover, we could expand NK cells from as little as 4 × 10^5^ PBMCs isolated from the blood sample of a breast cancer patient. Overall, we have observed that NK cells from breast cancer patients expand to a similar degree as healthy donors. Furthermore, it was possible to expand NK cells from breast cancer patient PBMCs which had been cryopreserved for up to a year. We used a panel of activation, maturation, and inhibitory markers to assess the phenotype of expanded NK cells from breast cancer patients. Our results suggest that the expansion of NK cells renders a specific NK cell phenotype regardless of the source of NK cell, with similar levels of activating, inhibitory, and maturation marker expression observed between healthy donor and breast cancer patient NK cells (Fig. 1c).

### Expanded NK cells from breast cancer patients have similar cytotoxicity against the TNBC cell line MDA-231/luc to those expanded from healthy donors

**Fig. 2:**
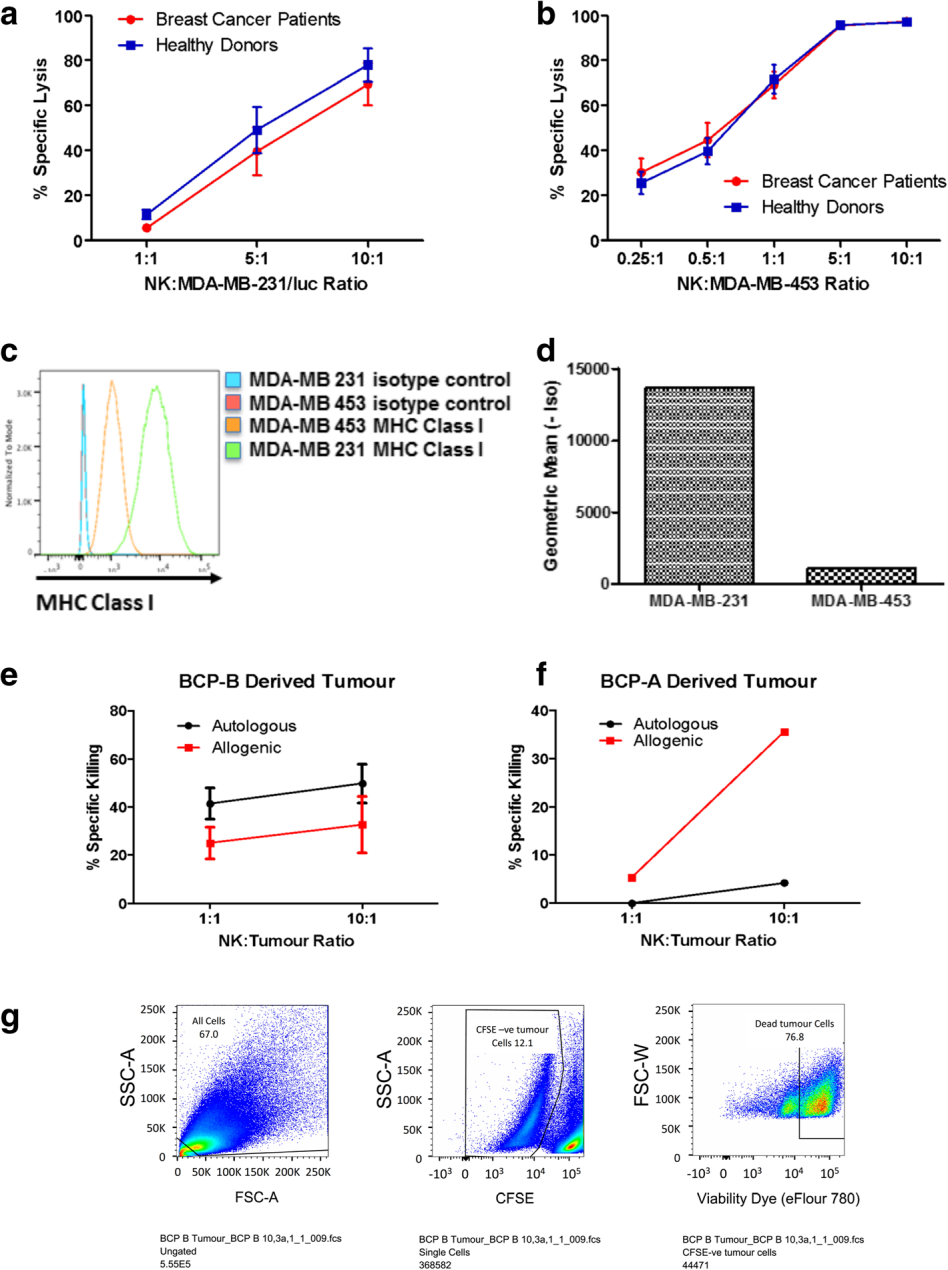
Expanded natural killer (*NK*) cells show high cytotoxicity against breast cancer cell lines and primary tumours in vitro. **a**, **b** Expanded NK cells from breast cancer patients (*red*) or healthy donors (*blue*) were plated with tumour cell lines at different NK cell to tumour cell ratios. After 4–5 h, cells were stained with viability dye and then percent cell death was assessed using flow cytometry. **a** NK cell cytotoxicity against the triple negative MDA-MB-231/luc cell line. **b** NK cell cytotoxicity against the HER2-positive MDA-MB-453 cell line. Percent-specific lysis was calculated and graphed (*n* = 4, mean ± SEM). Unpaired Student’s *t* test was used for statistical comparison between the groups. **c**, **d** MDA-MB-231 and MDA-MB-453 cells were stained for the expression of major histocompatibility complex (*MHC*) class I (anti-HLA-A,B,C–PE antibody) and assessed by flow cytometry. Results are shown in **c** histogram and **d** geometric mean. **e**–**g** Expanded NK cells from autologous breast cancer patient (*black*) or allogenic breast cancer patient (*red*) were carboxyfluorescein succinimidyl ester (*CFSE*)-labelled and plated with patient-derived breast cancer tumour cells at different NK cell to tumour cell ratios, and incubated and assessed as in **a** and **b**. Cells were gated on singlets, then tumour cells were selected using a CFSE negative gate (**e**) or in a hCD56 negative gate (**f**) and finally analysed for cell death. **e** Experiment was carried out using patient-derived tumour from breast cancer patient B (*BCP-B*), presenting with oestrogen receptor- and progesterone receptor-positive (ER^+^/PR^+^/HER2^–^) breast cancer. NK cells were CFSE-labelled prior to the assay. Data show the average from three independent experiments using the same patient-derived tumour sample. Unpaired Student’s *t* test was used for statistical comparison between the two groups. **f** Percent-specific lysis from one experiment carried out using patient-derived sample from breast cancer patient A (*BCP-A*) presenting with TNBC. NK cells were stained with hCD56. **g** An example of the gating used for **e**

It has already been established that the expansion process activates NK cells and increases their cytotoxic ability compared to those freshly isolated from peripheral blood. However, our study aimed to assess the functionality of NK cells expanded from breast cancer patients compared to those from healthy donors against breast cancer cell lines in vitro. To test for NK cell functionality, we assessed cytotoxic ability against the TNBC cell line MDA-MB-231/luc. Our results show that expanded NK cells from both healthy donors and cancer patients are highly cytotoxic against the MDA-MB-231/luc TNBC cell line (Fig. 2a).

### The HER2-positive breast cancer cell line is significantly more susceptible to NK cell killing than the TNBC cell line

We have already shown that the TNBC cell line MDA-MB-231/luc is highly susceptible to NK cell killing (Fig. 2a). However, we wanted to assess the ability of NK cells expanded from breast cancer patients and healthy donors against another breast cancer cell line. Thus, we tested their cytotoxicity against the HER2-positive breast cancer cell line MDA-MB-453. Similar to their cytotoxic activity against MDA-MB-231/luc, NK cells expanded from both breast cancer patients and healthy donors were highly cytotoxic against the HER2-positive cell line (Fig. 2b). Interestingly, the HER-2 positive cell line was significantly more sensitive to NK cell killing than the triple negative cell line. At a 1:1 ratio of NK cells to the tumour target cells, NK cells achieved an average of 70% specific lysis with MDA-MB-453 compared to 9% specific lysis with MDA-MB-231/luc (Fig. 2a and b). We further investigated the difference in susceptibility to NK cell killing and found it to be correlated with the degree of major histocompatibility complex (MHC) class I expression (Fig. 2c and d).

### NK cells expanded from breast cancer patients show cytotoxicity against autologous and allogenic patient-derived xenograft breast cancer cells

After observing that ex vivo expanded NK cells show very high cytotoxicity against breast cancer cell lines, we investigated the cytotoxicity of ex vivo expanded NK cells from breast cancer patients against both autologous and allogenic patient-derived breast cancer cells. Patient-derived tumour cells were obtained and processed as described previously. Expanded NK cells were labelled with CFSE and then incubated with patient-derived tumour cells, and cell death was analysed using flow cytometry (gating strategy is shown in Fig. 2g). The experiment was repeated three times with the same patient-derived sample (Fig. 2e). For one of the repeats, NK cells were not labelled with CFSE but instead were stained for hCD56 and gated out during tumour cell death analysis. For the sample from breast cancer patient A (Fig. 2f), tumour cells were stably passaged in vitro for 8 passages giving us enough cells to perform the cytotoxicity assay once. Our results show that patient-expanded NK cells showed cytotoxicity against both autologous and allogenic tumour cells in vitro.

### Expanded NK cells survive in vivo, in immunocompromised NRG mice, with the highest percentage of NK cells residing in the liver

**Fig. 3:**
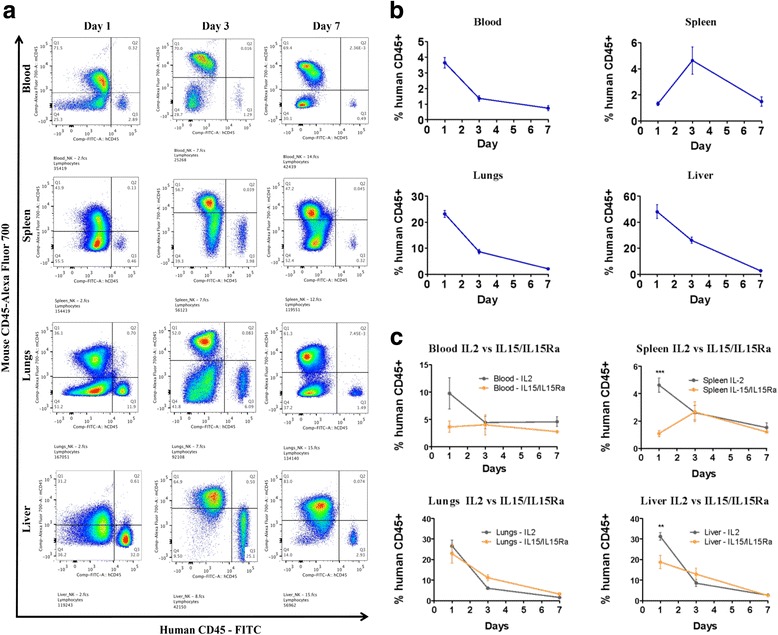
Number of expanded NK cells has steady decrease in vivo with interleukin (*IL*)-2 or IL-15/IL-15Rα injections. **a**, **b** Ten million expanded NK cells from a healthy donor were injected into NRG mice with daily IL-2 injections (20,000 Units). Mice were euthanized on days 1, 3, and 7. Blood, spleen, lungs, and liver were harvested and cells were isolated and stained with mCD45, hCD45, CD56, and CD3 antibodies and examined via flow cytometry. Percent human CD45^+^ cells were calculated based on total mCD45^+^ and hCD45^+^ cells and graphed. **a** Representative flow plots of three mice euthanized on days 1, 3, and 7. **b** Percent hCD45^+^ cells in each tissue was calculated and plotted (*n* = 5, mean ± SEM). **c** Similarly, 10 million expanded NK cells from a healthy donor were injected into NRG mice with daily injection of either 20,000 Units of IL-2 (*grey*) or IL-15 (500 ng) and IL-15Rα (1000 ng) (*orange*) (*n* = 3, mean ± SEM). Two-way ANOVA was used to compare the difference between both groups at the different time points, and a Bonferroni was used as a post-hoc test. **P < 0.01, ***P < 0.001

To evaluate the ability of NK cells to reduce tumour burden in mice, we had to first establish an appropriate schedule and dose of NK cell injection and determine whether we needed to administer cytokines in vivo to prolong NK cell survival/activation. Therefore, we assessed the survival of NK cells in vivo. We injected 10 million NK cells into NRG immunocompromised mice with daily IL-2 injection. We then euthanized the mice on days 1, 3, and 7 and collected blood, spleen, liver, and lungs. After tissue harvesting, we processed the tissues into single-cell suspensions and the cells were stained for mouse CD45 (mCD45), human CD45 (hCD45), human CD56, and human CD3. The percent human CD45 was calculated based on the total number of events from both human and mouse CD45-positive populations. This was done under the assumption that all mice would have similar levels of mCD45 cells. We also assessed the hCD45 population for the expression of CD56 and CD3. Our data showed that 94–98% of the hCD45^+^ population were NK cells, based on the CD56^+^CD3^–^ population. The data showed that NK cell survival has a steady decrease until day 7, where the percentage of NK cells was significantly lower (Fig. 3a and b). The NK cell population persisted in the mice at day 7, however, at a very low level. Interestingly, the liver had the highest percent of NK cells, 40–60% at day 1, compared to all other tissues (Fig. 3b). In the absence of IL-2 injections, the percentage of human NK cells in vivo, in immunocompromised mice, was very low and we could not detect NK cells past day 1 post-injection.

### IL-2 and IL-15 + IL-15Rα have the same effect on NK cell survival in vivo

We have shown that IL-2 is required to support survival of these NK cells in an immunocompromised mouse model. On the other hand, a high toxicity associated with IL-2 administration has been observed in clinical trials [40]. Moreover, IL-2 administration has been shown to also increase the percentage of circulating regulatory T cells in cancer patients, thus contributing to the inhibitory effects on NK cell cytotoxicity [41]. Another cytokine that can be used is IL-15; IL-15 is a cytokine that enhances the survival and cytotoxicity of NK cells [42, 43]. Additionally, the use of IL-15 in the clinic has shown promising results [36, 44]; therefore, we investigated the effects of IL-15 with IL-15Rα on the survival of expanded NK cells in vivo. Our results show no overall significant difference between the use of IL-2 and IL-15/IL-15Rα on prolonging the survival of NK cells in vivo (Fig. 3c). However, our results suggest that IL-2 might help with initial survival of NK cells in vivo. Thus, we decided to use IL-2 in the rest of our experiments to enhance NK cell survival.

### Expanded NK cells prevent the establishment and growth of the MDA-231/luc breast cancer cell line in a xenograft mouse model

**Fig. 4:**
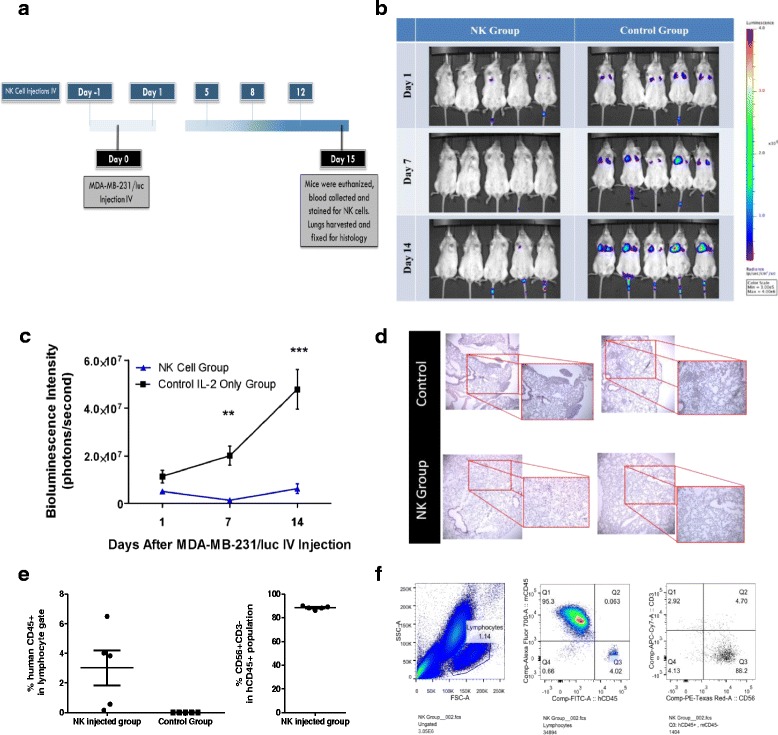
Expanded natural killer (*NK*) cells prevent the establishment and growth of tumour in a xenograft mouse model. **a** A group of five mice (the NK cell group) was injected IV with a dose of 10 million expanded NK cells at one day prior to tumour injection (day –1). At day 0, both groups (NK cell group and control group) were given a tumour injection of 0.5 × 10^6^ MDA-MB-231/luc cells IV. The NK cell group was given NK cells again at days 1, 5, 8, and 12. Both groups received daily IL-2 injections (20,000 Units). Mice were imaged using Xenogen IVIS Imager on days 1, 7, and 14. **b** Shows the bioluminescence pictures of the mice. **c** Total bioluminescence intensity (photons per second) was analysed and graphed. Two-way ANOVA was used to compare the two groups, and a Bonferroni was used as a post-hoc test to compare the difference between the two groups at a specific time point (*n* = 5, mean ± SEM). **P < 0.005, ***P < 0.0005. **d** Mice were euthanized on day 15 and lungs were collected and fixed in 10% formalin. Histology slides were then obtained and stained with haematoxylin and eosin stain. Shown are representative histology slides of the lungs of two control mice and two NK cell group mice. **e** Blood was collected from mice on day 15. Cells were stained with mCD45, hCD45, CD56, and CD3 antibodies and examined via flow cytometry. Percent human CD45^+^ cells were calculated based on total mCD45^+^ and hCD45^+^ cells and graphed. Percent of CD56^+^CD3^–^ NK cells within the hCD45 gate was graphed. **f** An example of gating: cells were gated on the lymphocyte gate, then the hCD45^+^mCD45^–^ gate, and finally on CD56^+^CD3^–^ cells

We then investigated the ability of expanded NK cells to prevent the establishment of tumours in a xenograft metastatic mouse model. We injected 10 million NK cells IV into five NRG mice at day –1. At day 0, mice received 5 × 10^5^ MDA-MB-231/luc, a TNBC cell line, IV with a daily dose of IL-2 (20,000 U). Our control group received the tumour cell injection with a daily IL-2 dose. The treatment group received another NK cell injection at day 1 and then twice a week (every 3–4 days) for another two weeks (Fig. 4a). At days 1, 7, 12, and 14 post-tumour injection, we imaged all 10 mice using Xenogen IVIS Imager (PerkinElmer) (Fig. 4b). At day 15, blood was collected and stained for mCD45, hCD45, human CD56, and human CD3. Percent human CD45 was calculated based on the total number of events from both the human and mouse CD45-positive populations (Fig. 4e). Based on CD56 and CD3 staining, 86–90% of the hCD45^+^ population isolated from blood of NK cell-injected mice were NK cells (Fig. 4e). The gating strategy used for the NK cell population is shown in Fig. 4f. At day 15 mice were euthanized and their lungs were collected and fixed to confirm our findings using histological staining (Fig. 4d). Our results show that expanded NK cells prevented tumour establishment and growth in the NK cell-treated group (Fig. 4b and c). There was a significant increase in tumour burden in the lungs of the mice in the control group compared to the NK cell-treated group (Fig. 4b). Moreover, histology of the lungs confirmed the results seen using IVIS (Fig. 4d).

## Discussion

Natural killer cells play a major role in cancer immunosurveillance. They are the first line of defence against transformed and virally infected cells [45]. However, when malignant cells escape the defences of the immune system, an immunosuppressive microenvironment is created [10–12]. The tumour secretes factors such as soluble MICA, MICB, and vesicles containing ULBP3, all of which impair NK cell cytotoxic ability, facilitating immune evasion [13, 14]. They also produce tumour cell-derived factors and tumour-derived exosomes, all of which suppress NK cell activity [46]. These factors cause decreased expression of NK cell-activating receptors while increasing the expression of inhibitory receptors [13, 14]. The goal of our study was to explore the use of autologous expanded NK cells as cell adoptive therapy against breast cancer by both increasing the numbers as well as cytotoxic ability of NK cells in breast cancer patients, allowing the suppressive microenvironment of the tumour to be overcome. Several studies have shown that NK cells isolated from cancer patients are characterized by decreased cytotoxicity, thus to be used in a clinical setting we first had to investigate the functionality of breast cancer patient NK cells post-expansion [8, 16]. It has been shown by others that expanded NK cells from healthy donors have higher cytotoxic ability than NK cells freshly isolated from peripheral blood [17, 34, 35]. To use autologous NK cells isolated from breast cancer patients as a cancer treatment, it was critical to demonstrate that the expansion process activates NK cells to similar levels as those observed in NK cells expanded from healthy donors. NK cells show variable cytotoxicity against different cancer cell lines [34]. However, it has been established by many groups that expanded NK cells show higher cytotoxic ability against tumours than NK cells freshly isolated from peripheral blood [17, 34, 35]. Thus, we investigated the ability of expanded NK cells to kill breast cancer cell lines in vitro. We showed that NK cells expanded from breast cancer patients effectively killed breast cancer cell lines. Though the cytotoxicity of breast cancer patient-derived NK cells had been demonstrated in vitro, their efficacy in an in vivo system has yet to be explored. We showed that NK cells can survive in vivo in immunocompromised mice for up to 3–4 days with either IL-2 or IL-15/IL-15Rα injections. The expanded NK cells were also able to prevent the establishment and growth of the MDA-231 triple negative breast tumour cell line in vivo in immunocompromised mice. Finally, using patient-derived tumour samples we demonstrated that both autologous and allogenic ex vivo expanded NK cells are cytotoxic towards patient-derived tumours.

**Fig. 5:**
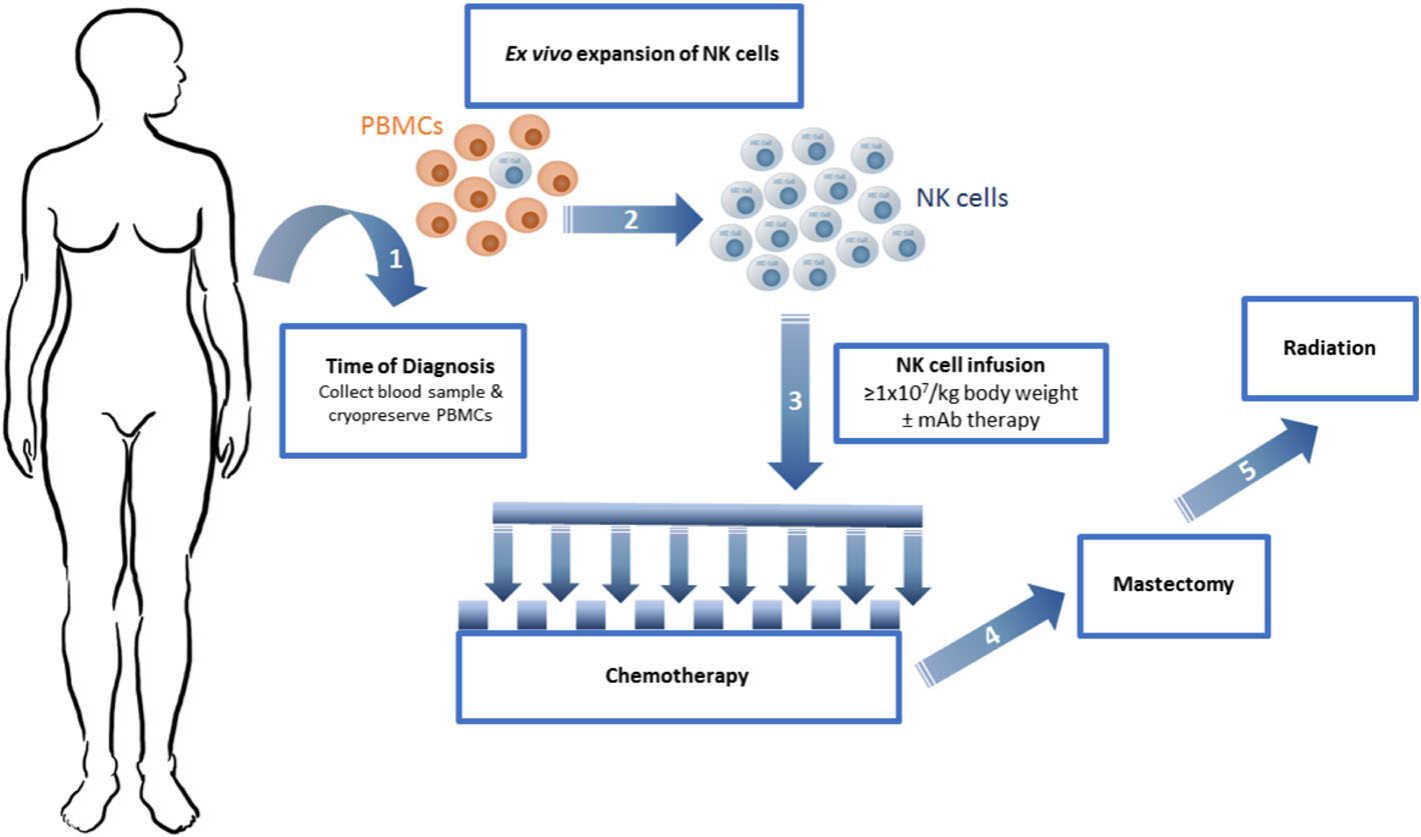
Restoration of natural killer (*NK*) cell function following chemotherapy. (1) At time of diagnosis, blood will be collected from the breast cancer patient and peripheral blood mononuclear cells (*PBMCs*) will be isolated and cryopreserved. (2) Four to five weeks prior to surgery, PBMCs will be thawed and ex vivo expansion will begin via co-culture with K562mbIL-21 aAPCs. (3) Two to three days post-chemotherapy, ex vivo expanded NK cells will be infused back into the patient at a dose of ≥1 × 10^7^/kg body weight. This infusion will be repeated 2 to 3 days after each round of chemotherapy at the same time point as monoclonal antibody (*mAb*) treatment such as anti-HER2 or anti-GD2 is delivered to the patient. After the last round of chemotherapy and NK cell infusion, (4) mastectomy would be performed followed by (5) radiation therapy

Our findings indicate that the NK cell expansion protocol described here can be applied to the clinic. We have demonstrated that PBMCs from breast cancer patients can be isolated at an early time point, such as at the time of diagnosis, and cryopreserved for later use without affecting their expansion ability or functionality. Further, the NK cells expanded from breast cancer patients are phenotypically and functionally similar to those expanded from healthy donors, showing a high level of cytotoxicity against both breast cancer cell lines and primary tumour. Here, we propose the following treatment schematic that incorporates the use of autologous NK cell therapy into the current standard-of-care treatment regime (Fig. 5). Upon diagnosis, peripheral blood would be collected from patients and PBMCs would be isolated and cryopreserved. Four to five weeks prior to surgery, NK cells would be expanded from PBMCs in a Good Manufacturing Practice (GMP) facility, in compliance with GMP standards. Infusion of NK cells would occur 2–3 days after each round of chemotherapy. Following this, mastectomy and radiation therapy would occur.

Delivery of NK cells in conjunction with chemotherapy has been shown to be safe and potentially effective [47]. NK cell infusion shortly after chemotherapy would act to repopulate the patient with their own, highly activated NK cells, and subsequently restore NK cell function. While the primary goal of chemotherapy is to decrease tumour size, it is also known that chemotherapy affects immune cell populations and tumour cell surface marker expression [48]. The increased expression of cancer antigens and immunogenic molecules induced by chemotherapy on tumour cells could be favourable for the function of the infused NK cells. In addition, increased apoptosis in immunoregulatory cells, such as regulatory T cells and MDSCs, has also been observed following chemotherapy, which would better support NK cell activation [48].

Another approach which could be delivered in conjunction with NK cell immunotherapy is monoclonal antibody therapy. Combining NK cell adoptive therapy with monoclonal antibodies against tumour-specific antigens could provide NK cells with higher specificity against tumour cells. For example, a standard therapy for HER2-positive breast cancer is the anti-HER2 antibody trastuzumab, whose main mechanism of action is through antibody-dependent cellular cytotoxicity by interacting with Fc receptors, such as CD16 expressed on NK cells [49–51]. While NK cells independently have the ability to recognise and kill tumour cells, the use of antibodies against tumour surface antigens could highly increase the specificity of NK cells to their tumour targets. Another potential cancer therapy that has gained major interest in the past decade is the use of immune checkpoint inhibitors [52]. The use of antibodies against immune checkpoint molecules such as programmed cell death protein 1 (PD-1), expressed mainly on T cells and NK cells, has shown promising responses against many cancers. Recently, it has also been shown that the use of anti-PD-1 can enhance NK cell cytotoxicity against tumour cells in vitro and in vivo [53].

The combination of NK cell infusion with radiation therapy is another option that could also enhance the efficacy of NK cell therapy. Ionizing radiation, for example, enhances expression of NKG2D ligands on tumour cells, thus rendering them highly susceptible to NK cell killing [54, 55]. Thus, the use of NK cell adoptive therapy has the potential to be used in combination with multiple therapies to enhance efficacy without eliminating or hindering the current standard-of-care treatments.

While we examined just one of many potential cell therapy approaches to treating cancer, another area that has garnered much interest is the use of chimeric antigen receptor (CAR) T cells. In this treatment strategy, T cells are manipulated to express a CAR specific for a tumour-associated antigen, allowing T cell cytotoxicity to be directed against tumours independently of MHC class I antigen presentation. The emergence of CAR T cells has been a great clinical success in the field of cancer immunotherapy [56, 57]. Indeed, the robust killing and high level of specificity associated with CAR T cells has proven successful, particularly against B cell malignancies by targeting the B cell marker CD19. Despite these early successes with CAR T cells, several limitations have impeded their use. One such limitation is due to the non-discriminatory nature of CAR T cell activation in response to healthy, off-tumour targets [58, 59]. NK cells, on the other hand, respond to a balance of activating and inhibitory signals which prevent NK cell cytotoxicity from being directed against healthy tissues [60]. Indeed, the infusion of NK cells has been shown to be safe and well tolerated by patients [24, 32, 61].

Due to unrestricted activation of CAR T cells in response to antigen expression on healthy tissues, antigen targets for CAR T cell therapies must be selected for those that are expressed exclusively on the tumour, or in the case of CD19-specific CAR T cells for the treatment of B cell lymphomas, expressed on a single cell lineage [62, 63]. Use of NK cells, however, does not require engineering or targeting of a specific antigen. For CAR T cells, this causes further issues both in the discovery of such antigens and also in the context of antigen loss on the tumour [57, 64]. Unlike CAR T cells, NK cell activation and cytotoxicity is not reliant on specific interactions with tumour-associated antigens and as a result they are not impacted by tumour heterogeneity and antigen loss on the tumour. Furthermore, NK cells are able to direct their cytotoxicity towards tumour cells via multiple mechanisms including antibody-dependant cellular cytotoxicity through the CD16 NK cell receptor. This makes NK cells an ideal candidate for combination with antibody therapies, such as the use of the HER2-specific antibody trastuzumab [65]. Overall, while CAR T cells are capable of very robust killing of tumour cells and their application to certain cancers, such as B cell malignancies, has resulted in very promising results, the safety of NK cell delivery, their ability to elicit cytotoxic effects both directly and via antibody-mediated cellular cytotoxicity, and their efficacy in the context of antigen loss make them an attractive option for new cancer therapies.

## Conclusion

Our data provide evidence that NK cells can be expanded from breast cancer patients. Moreover, these ex-vivo expanded NK cells show high levels of in vitro cytotoxicity against breast cancer cell lines and both autologous and allogenic patient-derived tumour cells. However, further investigation is required to provide data about the ability of expanded NK cells to treat patient-derived tumour cells in vivo.

## Abbreviations

(a)APC: (Artificial) antigen-presenting cell
BCP: Breast cancer patient
CAR: Chimeric antigen receptor
CSFE: Carboxyfluorescein succinimidyl ester
ER: Oestrogen receptor
FBS: Fetal bovine serum
hCD45: Human CD45
HD: Healthy donor
HER2: Human epidermal growth factor receptor 2
IL: Interleukin
IV: Intravenously
KIR: Killer-cell immunoglobulin-like receptor
mCD45: Mouse CD45
MDSC: Myeloid-derived suppressor cell
MHC: Major histocompatibility complex
NCR: Natural cytotoxicity receptor
NK: Natural killer
NRG: NOD-Rag1^–/–^-γ^–/–^
PBMC: Peripheral blood mononuclear cell
PBS: Phosphate-buffered saline
PD-1: Programmed cell death 1
PR: Progesterone receptor
RMF: Reduction mammary fibroblasts
TNBC: Triple negative breast cancer
